# Review on the effect of systemic melatonin in periodontal disease

**DOI:** 10.6026/97320630019692

**Published:** 2023-06-30

**Authors:** Iram Rafique Pawane, Sankari Malaiappan

**Affiliations:** Department of Periodontics, Saveetha Dental College and Hospitals, Saveetha Institute of Medical and Technical Sciences, Saveetha University, Chennai - 600077, India

**Keywords:** Chronic periodontitis, melatonin, systematic review, evidence based dentistry, periodontitis

## Abstract

It is of interest to determine the efficacy of adjunctive systemically administered (via oral route) melatonin compared to a placebo, on changes in clinical parameters in patients with periodontitis undergoing periodontal therapy. Literature search was performed systematically after searching with 4 electronic databases until December 2022. Primary outcome measures were changes in Probing Pocket Depth (PPD) and bleeding on probing (BOP%). Secondary outcome measures taken were Clinical attachment Level (CAL) gain and level of Tumor Necrosis Factor-alpha (TNF-α). Risk of bias analysis was done for all the studies. With the adjunctive use of melatonin, improved probing depth reduction and gain in clinical attachment levels was observed in all four studies and was found to be statistically significant compared to the placebo group, suggesting that melatonin administered systemically was able to exert a positive effect at diseased sites. Melatonin drug administered at 10 mg dosage for 2 months showed a significant decrease in TNF-α levels; but showed no significant difference between TNF-α levels in the test and control group at 6 mg administration for 2 months. Systemically administered melatonin as an adjunct in treating periodontal disease could potentially be used due to its antioxidant, anti-inflammatory and bone remodelling properties from increasing evidence. However, a lack of proper guidelines, side effects, standardization of dosage for treating periodontitis and its long-term effect on various other systems in the body, patient related outcome factors and the effect of increased dietary melatonin efficacy have yet to be taken into consideration. Due to the lack of homogeneity, differences in dosage uniformity, and varying follow up intervals, more randomized controlled trial protocols are required to fill the lacunae and better improve our understanding.

## Background:

Periodontitis is a chronic inflammatory condition which is the sequel of multiple bacterial and host factors previously considered to be solely pathogen induced. [1] Periodontitis occurs due to host-pathogen dysbiosis which consists of uncontrolled immune responses, destruction of the periodontium and surrounding structures with the release of reactive oxygen and nitrogen species and enzyme imbalances. [2] To treat these, various adjuncts have been introduced alongside conventional therapy of scaling, root planing and flap surgery; such as hyperbaric oxygen therapy, lasers, chemical and antimicrobial agents, etc. [3-see PDF] Treating periodontal disease encompasses mechanical non-surgical and surgical periodontal therapy, however this is not sufficient to eliminate the bacterial toxin load and ensure inflammation resolution in the periodontal pocket. [4] To enhance the results of mechanical therapy for the resolution of the signs and symptoms of periodontitis, the use of systemic, local and topical adjuncts have emerged. [5]

One such agent being used as an adjunct to periodontal disease is the hormone melatonin. This endogenous hormone is produced by the pineal gland, which is present in the midline of the brain and attached at the roof of the third ventricle and is under the control of the supra-chiasmatic nucleus. [6] Melatonin are also secreted by extra pineal organs such as the retina, spleen, bone marrow, testicles, ovaries, etc. and does not act on a particular target organ, reaching all cellular components such as the mitochondria and nucleus due to its lipophilic properties. [7] Thus, melatonin plays a key role in physiological processes such as circadian rhythm regulation, immune function, ovarian physiology etc.

The antioxidant properties of melatonin have been widely studied. Melatonin and its metabolites are potent free radical scavengers and activate mechanisms that stimulate enzymes that have antioxidant activity. [8] Other than being a powerful antioxidant, melatonin has a protective role against the oxidation of proteins, lipids and DNA damage as it is concentrated in the mitochondria. [9,10] Stimulation of transcription activity and inhibition of the formation of the hydroxyl free radical are also some of the properties of the indoleamine hormone. [11],[12, 13] The investigation of the role of melatonin and its applications in the regeneration and remodeling of bone is currently being researched with respect to osteoporosis in females and some studies have reported its role in osseointegration of implant dentistry. [14],[15]

Melatonin hormone minimizes bone resorption by reducing levels of reactive oxygen species (ROS) and receptor activator of nuclear factor kappa beta ligand (RANKL) which stimulates osteoclastogenesis and oxidative stress within the tissue and cause local destruction. Melatonin also simultaneously promotes new bone regeneration by enhancing the proliferation, migration, chondrogenic and osteogenic differentiation of mesenchymal stem cells, along with the action of increasing the vascular endothelial growth factor (VEGF) levels, enhances angiopoiesis at sites of bone resorption, leading to the promotion of healing and prevention of ischemic injuries.[16,17] As the role of melatonin as an adjunct in treating periodontal disease has gained momentum, the efficacy of systemically administered melatonin on periodontal disease outcomes are not well understood. Therefore, the purpose of this systematic review was to assess how patients with periodontal disease responded to systemic melatonin.

## Materials and Methods:

According to the PRISMA (Preferred Reporting Items for Systematic Reviews and Meta-Analyses) guidelines, this systematic review was created. [18]

##  Focused questions:

The two structured questions below were the focus of the current review:

[1] In patients with periodontitis, do systemically administered melatonin (compared to placebo) influence clinical periodontal parameters?

[2] In patients with periodontitis undergoing adjunctive systemic melatonin (compared to placebo) is there a change in inflammatory mediators?

## Eligibility criteria:

We used the PICOS framework to arrive at the above focused questions:

Population (P): systemically healthy patients with periodontitis undergoing periodontal therapy

Intervention (I): For the Test group, an adjunctive systemically administered Melatonin (via oral route) supplement.

Comparisons (C): control group received a placebo

Outcomes (O):

i. Primary outcome measures: Probing Depth reduction (PD), Clinical Attachment Level (CAL) gain.

ii. Secondary outcome measures: Bleeding on Probing scores (BOP) and proinflammatory marker levels.

Study designs (S): This systematic review only included Randomized Placebo Controlled Clinical Trials.

## Inclusion criteria and exclusion criteria:

i. Patients with periodontitis undergoing non-surgical periodontal therapy indicated for flap surgery.

ii. Adjunctive administration of systemic melatonin via oral route as well as placebo controlled parallel design studies.

iii. Studies published in the English language.

iv. Human trials with ethical clearance

Studies were excluded if they were animal studies, literature reviews, studies recruiting less than 30 patients and melatonin administered via topical application (mouthwashes, gels), subgingival delivery etc.

## Information sources and search:

To find relevant studies for the review, a skilled reviewer (S.M.) searched electronic databases up until December 2022. Original research papers were searched on:

[1] The National Library of Medicine (MEDLINE by PubMed)

[2] The Cochrane Database Trials Register

[3] GOOGLE SCHOLAR and

[4] SCIENCE DIRECT.

**Figure 1:**
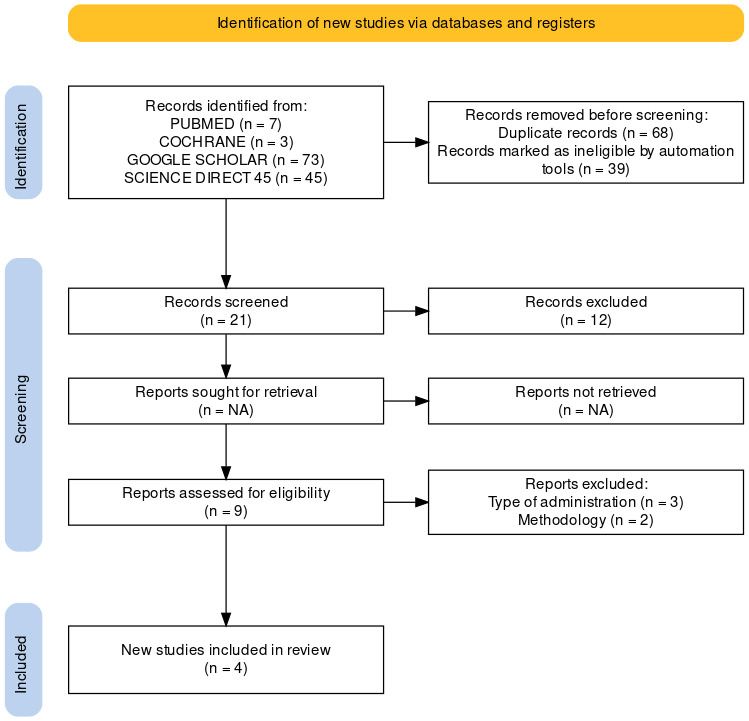
PRISMA flow diagram of included studies.

Figure 1 represents the 2020 PRISMA flow diagram for the included studies in this review. [19]

## Data collection process and data items:

All studies that met the inclusion criteria were then subjected to quality review and data collection. The total number of patients, demographic groups, and diagnosis, clinical and radiological outcomes and follow-up period were all recorded on a standardized, uniquely designed data extraction form for each included study. Two review authors (I.R. and S.M.) independently extracted data.

## Study characteristics:

The systematic review only included RCTs with a placebo control. At the follow-up visit, CAL gain had to be expressed as the mean increase in millimeters of the treated sites in each arm of the trial. At the follow-up visit, PD reduction had to be expressed as a mean decrease in millimeters in periodontal probing depth at the treated sites.

## Risk-of-bias analysis

Two review authors independently evaluated the included studies' quality using a risk of bias analysis which might have an impact on the overall inferences ("Systematic reviews, CRD's guidance for undertaking reviews in health care," University of York, 2008). For determining bias risk, the Cochrane Collaboration's tool was utilized. [20] In a specific table, seven domains were considered, including sequence generation, allocation concealment, blinding of participants and reviewers, blinding of the outcome appraiser, incomplete outcome data, selective outcome reporting, and other bias. The included studies' risk of bias was categorized as follows:

If all requirements were met, there was a low risk of bias.

If one or more criteria were partially met, there was an unclear risk of bias.

**Figure 2:**
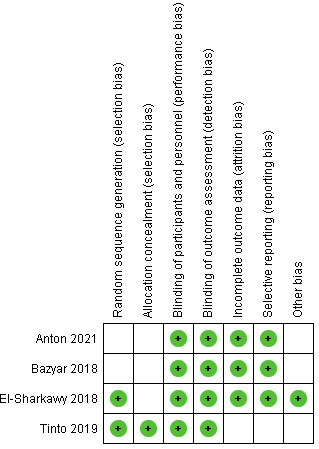
Risk of bias analysis of the included studies

High risk of bias if one or more criteria were not met (Figure 2).

## Evaluation of the strength of evidence:

A single reviewer (I.R.) performed the statistical analyses using the RevMan software version 5.3 (Copenhagen: The Nordic Cochrane Centre, The Cochrane Collaboration, 2014). Different levels of methodological strength modified from GRADE (Grading of Recommendations Assessment Development and Evaluation) were used to rate the studies.

There were three levels of evidence strength considered:

High: At least three RCTs with low heterogeneity and bias risk.

Moderate: More than one RCT and at least one RCT with low bias risk and low heterogeneity.

**Figure 3:**
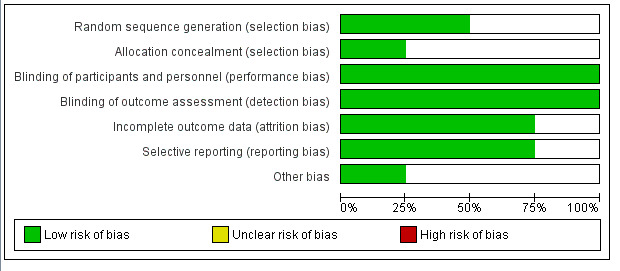
Risk of bias analysis of the included studies.

Low: No RCTs, RCTs with high bias risk, or RCTs with significant heterogeneity (Figure 3)

## Results:

## Study selection:

The search in databases of PUBMED, Cochrane, Google Scholar and Science Direct provided 128 articles published until December 2022. After title screening and removing duplicate studies, 21 articles were filtered out. After reading the full texts of all 21 articles, 4 articles were chosen as meeting all the requirements for inclusion in this systematic review, and 17 articles were left out of the analysis.

## Study characteristics:

All four randomized controlled clinical trials were placebo controlled parallel design studies. [21,22, 23, 24] Three studies were double blinded that were conducted in university settings, one in Egypt, Iran and Switzerland.[21,22, 24] One out of the four studies was triple blinded in a private setting in a single center.[23] The duration of each of the studies ranged from 1-2 months which used placebo as a control and hormone melatonin as 10mg, 6mg, and 1 mg tablets administered systemically one time a day before bed. 192 subjects are taken into consideration in this review.

## Synthesis of results:

The results from the review for the clinical parameters and biological markers were assessed and are follows:

## Probing depth

Probing depth was evaluated in the 4 clinical trials. Statistically significant reduction in probing depth was observed in all the studies (p<0.001) the best results demonstrated at 6 months postoperatively.

## Clinical Attachment Level (CAL)

Out of four included clinical trials, CAL was recorded in 3 of the studies (El-Sharkawy *et al*., Bazyar *et al*. and Anton *et al*.). Each of the trials reports a statistically significant gain in CAL gain over a period of 2-6 months.

## Bleeding On Probing (BOP)

BOP was recorded in 3 out of the four trials: El-Sharkawy *et al*. as BOP%, Tinto *et al*. as FMBS% (Full Mouth Bleeding Score%) and Anton *et al*. as BOP). Neither of the studies reported a significant difference in bleeding index before and after intervention with systemic melatonin.

## Proinflammatory marker levels

TNF-αis a cytokine produced during the cascade of inflammation by monocytes or macrophages responsible for various signaling events that lead to cell necrosis or apoptosis. It could also be a marker for inflammation. Out of the 4 studies taken for this review, 2 studies have included TNF-αas an outcome variable (El-Sharkawy *et al*. and Bazyar *et al*.). It was observed that, over the duration of 2 months, El-Sharkawy *et al*. reported a stark difference in TNF-αlevels in patients in the melatonin group (10mg) compared to placebo at follow-up (3 and 6 months). However, Bazyar *et al*. reported no significant changes in the test (6mg) and the control (placebo) groups at follow-up.

## Discussion:

To the best knowledge of the researcher, this is the first review created to assess the effectiveness of systemically administered melatonin as an adjunct in treating periodontal disease. Only randomized, placebo controlled, blinded clinical trials were considered for this review for the purpose of including evidence that is reliable for the clinician in choosing this drug for the treatment of periodontal disease, based on the RoB tool, most of the studies included in this review have a low risk of bias.

Two out of four studies included in this systematic review report a follow up period of 6 months from baseline (El-Sharkawy *et al*. and Tinto *et al*.). However, the other two studies report a follow up of 8 weeks (2 months) from baseline (Bazyar *et al*. and Anton *et al*.). This must be taken into consideration as the administration of the drug in three studies was given for a total duration of two months; but the study by Tinto *et al*. administered 1 mg melatonin for a duration of 1 month. This brings us to the challenge of interpreting the efficacy of the drug at concentrations of 1 and 10 mg and durations of 1 and 2 months respectively. Clinical parameters assessed in this review were Probing Depth reduction, Clinical Attachment Level gain and Bleeding on Probing. From the results of the above review, after one-stage full mouth NSPT and use of melatonin tablets, improvement in probing depth level and gain in CAL was observed in all four studies; statistically significant values were seen compared to the placebo group, suggesting that melatonin administered systemically was able to exert a positive effect at diseased sites.

Increased levels of inflammatory mediators in periodontitis patients have been linked to attachment loss and tissue damage via the expression of matrix metalloproteinase (MMP) and receptor activator of nuclear factor kappa-B ligand (RANKL) pathways. These mediators include interleukins (IL-1, IL6), prostaglandins, C-reactive protein, and tumor necrosis factor- (TNF-).[[25, 26, 27] To target the host response and prevent further tissue destruction due to own cells undergoing necrosis and cell death due to the inflammatory response, host modulatory agents such as COX inhibitors (NSAIDS), bisphosphonates, tetracyclines etc. were introduced. However, there is a significant disparity in host modulatory agents due to the high rate of recurrence and unfavorable side effects of long-term use of these drugs. Melatonin shows promise as it has shown potent antioxidant, anti-inflammatory, and cytoprotective properties. In the above review in the study by EL-Sharkawy *et al*. melatonin drug administered at 10 mg dosage for 2 months showed a significant decrease in TNF-αlevel; however, there was no significant difference between TNF-αlevels in the test and control group when 6 mg melatonin was administered for 2 months.

Despite evidence supporting the benefits of incorporating melatonin in periodontal therapy, some studies have reported no statistical improvement in periodontal parameters with or without intervention. One report demonstrated that the use of systemic melatonin did not reduce inflammatory markers in saliva, nor did it prevent bone loss in rats. In the same report, a mouth rinse formulated with melatonin reported adverse effects in patients with periodontal disease. [28] However, discrepancies in either study could occur in the dosage, use, and follow up period, sample size included in the study and patient compliance to usage of the drug over a long period of time.

Another limitation of the above review is that none of the studies report patient related outcome measures after the trial was completed. Melatonin, a circadian rhythm regulator, could influence the sleep-wake cycle if taken during the day and not at prescribed intervals. The dosage and time interval of the administration of the drug has not yet been determined for the use in periodontal therapy. Another point to be taken into consideration to design trials that would really highlight the role of the efficacy of using melatonin is the intake of melatonin from diet, circadian rhythm disorders, matching subject demographics (age, sex, lifestyle etc.). Although, from the present review, a 6mg formulation over a period of two months does show promise as an adjunct. More long-term clinical trials with uniform dose administration and larger sample size are the need of the hour.(Table 1)(Table 2)

## Conclusion:

Systemically administered melatonin as an adjunct in treating periodontal disease could potentially be used due to its antioxidant, anti-inflammatory and bone remodeling properties from increasing evidence. From the above review, due to lack of homogeneity, and dosage differences, and short-term follow-up, it is still unclear whether the use of melatonin has any beneficial role in treating periodontal disease. Additionally, a lack of proper guidelines, side effects, standardization of dosage for treating periodontitis and its long-term effect on various other systems in the body, patient related outcome factors and the effect of increased dietary melatonin efficacy have yet to be taken into consideration.

## Figures and Tables

**Table 1:** Properties of randomized controlled trials included.

**S No.**	**Author and Year**	**Study Design**	**Blinding**	**Setting**	**Parameters Assessed**	**Sample size and Groups**	**Dosage**	**Duration**
1	El-Sharkawy *et al*. 2018	Randomized Placebo Controlled Clinical Trial	Double blinded parallel design	Single center, University setting, Egypt	CAL gain, PD reduction, BOP (%), salivary TNF- α, AIS	74 Subjects with primary insomnia with periodontitis. 38 test subjects, 36 placebo-controlled subjects	10mg oral melatonin vs placebo (OD)	2
2	Bazyar *et al*. 2018	Randomized Placebo Controlled Clinical Trial	Double blinded parallel design	Single center, University setting, Iran	CAL gain, PD reduction, BOP (%), salivary TNF- α, IL_6, hs-CRP	44 type 2 diabetic subjects with periodontitis. 22 test subjects, 22 placebo controls	6mg (2 tablets of 3mg each) oral melatonin vs Placebo (OD)	2
3	Tinto *et al*. 2019	Randomized Placebo Controlled Clinical Trial	Triple blinded parallel design	Single center, Private setting, Italy	PD, Full Mouth bleeding Scores (FMBS%) (FMPS %)	20 systemically healthy patients stage III periodontitis. 10 test subjects, 10 placebo control	1 mg oral melatonin vs placebo (OD)	1
4	Anton *et al*. 2021	Randomized Placebo Controlled Clinical Trial	Double blinded parallel design	Single center, University setting, Switzerland	CAL gain, PD reduction, BOP (%), HbA1c, Plaque Index	54 type 2 diabetic patients. 27 test subjects 27 placebo controls	6mg (2 tablets of 3mg each) oral melatonin vs Placebo (OD)	2
CAL= Clinical Attachment Level
PD=Probing Depth, BOP=Bleeding on Probing
TNF-α=Tumor Necrosis Factor- α
AIS=Athen's insomnia score
IL-6=Interleukin-6
hs-CRP=high sensitivity C reactive protein
FMBS=Full Mouth Bleeding Score
FMPS=Full Mouth Plaque Score
HbA1c= Glycated Hemoglobin

**Table 2:** Outcomes of included studies

**Sr. No**.	**Author**	**Follow up period**	**Outcome**
1	El-Sharkawy*et al*. 2018	Baseline, 3, 6 months	Statistically significant difference in CAL gain, PD reduction at 3 and 6 months (p<0.01), TNF- α levels significantly lower in test compared to placebo groups, no significant differences in BOP% between both groups
2	Bazyar*et al*. 2018	Baseline, 8 weeks	Statistically significant differences between control and test group in IL-6, hc-CRP, PD and CAL values (p<0.001), no significant difference between test and control group in TNF-α levels
3	Tinto *et al*. 2019	Baseline, 6 months	No significant change seen between groups with respect to FMBS% and FMPS%. significant difference seen in PD reduction in test compared to control group
4	Anton *et al*. 2021	Baseline, 8 weeks	Significant improvement in PD reduction, CAL gain in test group compared to placebo. No differences seen in BOP, Plaque Index and HbA1c values.
CAL= Clinical Attachment Level
PD=Probing Depth, BOP=Bleeding on Probing
TNF-α=Tumor Necrosis Factor- α
AIS=Athen's insomnia score
IL-6=Interleukin-6
hs-CRP=high sensitivity C reactive protein
FMBS=Full Mouth Bleeding Score
FMPS=Full Mouth Plaque Score
HbA1c= Glycated Hemoglobin
